# Visual outcomes in patients with meningiomas compressing optic nerve

**DOI:** 10.3389/fneur.2025.1606661

**Published:** 2025-06-13

**Authors:** David Krahulik, Filip Blazek, Martin Hampl, Lumir Hrabalek, Jan Krahulik, Marta Karhanova

**Affiliations:** ^1^Department of Neurosurgery, University Hospital Olomouc, Olomouc, Czechia; ^2^Department of Neurosurgery, University Hospital Ostrava, Ostrava, Czechia; ^3^Department of Ophthalmology, University Hospital Olomouc, Olomouc, Czechia

**Keywords:** visual impairment, optic nerve compression, visual function prognosis, optical nerve, meningiomas

## Abstract

**Background:**

Meningiomas compressing the optic nerve can lead to progressive visual loss due to the nerve’s complex intraorbital, intracanalicular, and intracranial anatomy. Although observation, radiation, and surgical decompression are available, optimal strategies for preserving vision remain controversial. This study retrospectively evaluates the impact of surgical intervention on visual recovery in patients with optic nerve–compressing meningiomas to refine patient selection and treatment strategies.

**Methods:**

A retrospective review was conducted on medical records from the Neurosurgical Clinic at Olomouc University Hospital for patients undergoing surgical treatment for meningiomas near the optic nerve from 2015 to 2023. Inclusion criteria required high-quality preoperative and postoperative MRI, complete ophthalmic records, and a minimum one-year follow-up. Data on demographics, tumor characteristics (size, location, and relationship with the optic nerve), and visual function (acuity and field) were collected. Tumors were categorized by size and degree of optic nerve involvement, and visual outcomes were assessed pre- and postoperatively.

**Results:**

Seventy-nine patients (66 females, 13 males; mean age 58) met inclusion criteria. A longer duration of visual impairment correlated with more severe preoperative vision loss. Although postoperative visual function did not significantly correlate with tumor size, location, or duration of preoperative symptoms, patients with shorter impairment durations demonstrated better postoperative recovery.

**Conclusion:**

The duration of preoperative visual impairment is a critical predictor of visual outcomes, supporting early surgical intervention for optic nerve–compressing meningiomas. While tumor size and location did not directly influence recovery, further investigation into tumor-anatomy relationships is warranted to optimize visual prognosis.

## Introduction

Meningiomas located in the anterior and middle cranial fossae can exert pressure on the optic nerve (ON), resulting in varying degrees of visual impairment. The optic nerve is anatomically divided into three segments—intraorbital, intracanalicular, and intracranial—each of which influences the severity and pattern of compression. Intracranial meningiomas, particularly those arising in regions such as the olfactory groove or anterior clinoid process, may affect both the optic nerve and the optic chiasm, leading to a spectrum of symptoms that vary according to the specific tumor location and the anatomical configuration of the chiasm (1, 2).

Early manifestations of visual impairment are frequently observed in cases with prefixed chiasms, where the optic nerve is in closer proximity to the tumor, while patients with postfixed chiasms tend to exhibit a delayed onset of symptoms (3, 4). Diagnostic challenges arise from the complex anatomy of the intracranial space and orbit, areas that lack the dense supportive tissues found elsewhere, thereby complicating early detection. In contrast, meningiomas that extend into the optic canal (OC) often produce symptoms earlier in the disease course, prompting more rapid clinical evaluation. A detailed understanding of the optic nerve’s anatomical relationships with surrounding structures is therefore crucial for enhancing diagnostic accuracy and assessing visual risks based on tumor location (3, 5, 6).

Management strategies for these patients range from observation and radiation therapy to surgical excision, with the overarching goal of preserving or improving visual function (7). The surgical approach, particularly decompression of the optic canal via procedures such as unroofing, remains a subject of debate due to the inherent risks and the variable outcomes reported in the literature (8). For patients with smaller tumors or minimal visual impairment, conservative management through observation is often considered safe. In cases where more aggressive treatment is warranted, radiation therapy may serve as a less invasive alternative to surgery, particularly for tumors situated near critical brain structures (9, 10).

This study is designed to elucidate the influence of surgical interventions on visual outcomes in patients with optic nerve–compressing meningiomas. By analyzing a range of ophthalmic, imaging, and surgical predictors, we aim to identify key factors that correlate with improved prognosis. Our goal is to optimize treatment strategies to better preserve or restore vision in this patient population.

## Methods

The medical records from the Neurosurgical Clinic at Olomouc University Hospital were retrospectively reviewed to identify patients who underwent surgical treatment for meningiomas located near the optic nerve between 2015 and 2023. Inclusion criteria were limited to patients who had surgery at our clinic and had high-quality preoperative and postoperative MRI scans, along with complete ophthalmic records both before and after surgery. On MRI, optic nerve compression was verified by evaluating signs such as narrowing of the nerve, increased T2 signal intensity within the nerve, or other structural changes indicative of compression. All included patients had a minimum follow-up period of one-year post-surgery, with visual function assessments conducted at the Ophthalmology Clinic of University Hospital Olomouc.

Data collected from this cohort included demographic information, preoperative tumor characteristics such as tumor size, origin, its interaction with the optic nerve or optic chiasm. Additional variables considered included preoperative and postoperative measures of visual acuity and visual fields. The study was conducted in accordance with the Declaration of Helsinki and approved by the Institutional Review Board of University Palacky, Olomouc.

In total, 79 patients met the inclusion criteria for the study, consisting of 66 females and 13 males, with a mean age of 58 years. Tumor size was categorized into three groups by the maximum diameter in cm: (1) small tumors, under 3 cm; (2) medium tumors, ranging from 3 to 5 cm and (3) large tumors, over 5 cm. Based on the tumor’s relationship with the optic nerve or chiasm, the cohort was divided into four groups: (1) intimate contact with the optic nerve or chiasm, (2) compression of the optic nerve or chiasm, (3) dislocation of the optic nerve, and (4) encasement of the optic nerve within the tumor mass.

Tumor origin was classified into four categories: (1) planum sphenoidale meningiomas, (2) anterior clinoid meningiomas, (3) sphenoorbital meningiomas, and (4) cavernous sinus meningiomas. Meningiomas were categorized based on their primary site of origin and anatomical extension. For instance, if the tumor predominantly extended to the planum sphenoidale, it was classified under that group, while those extending primarily toward the anterior clinoid were included in the anterior clinoid category. The surgical approach varied depending on the tumor type and its anatomical relationship with surrounding structures, which likely influenced surgical outcomes and postoperative recovery.

Visual acuity and visual field assessments were recorded preoperatively, with patients categorized into three groups based on their visual status: (1) intact vision, (2) impaired vision, and (3) blindness. The term “visual status” encompasses both visual acuity and visual field deficits, as both aspects significantly impact quality of life in this disease. Postoperative visual outcomes were divided into four categories: (1) intact vision, (2) stable vision, (3) improved vision, and (4) worsened vision. These categories consider both visual acuity and visual field outcomes, as field deficits are common in this disease and significantly impact quality of life.

Statistical analysis was performed by a statistician. Categorical variables were compared using the chi-square test or Fisher’s exact test when appropriate. Continuous variables were assessed for normality, and comparisons between groups were made using either the ANOVA or Kruskal–Wallis test based on distribution. A receiver operating characteristic (ROC) curve was constructed to evaluate the relationship between symptom duration and postoperative visual outcome. A *p*-value < 0.05 was considered statistically significant.

## Results

### Patient population and tumor characteristics

Seventy-nine patients (66 females, 13 males; mean age 58 years) were evaluated using high-resolution imaging and comprehensive ophthalmologic examinations. Tumors were grouped by size: 13 patients had tumors smaller than 3 cm, 15 patients had tumors between 3 and 5 cm, and 5 patients had tumors larger than 5 cm. A descriptive trend was observed in which smaller tumors were generally associated with a shorter duration of visual impairment.

### Location

The most common tumor group was sphenoorbital meningiomas, with 25 patients, followed by cavernous sinus meningiomas (21 patients), anterior clinoid meningiomas (19 patients), and planum sphenoidale meningiomas (14 patients). The anatomical positioning of the tumors did not appear to directly affect visual acuity or visual field deficits in a statistically meaningful way. This suggests that other factors, such as tumor size, the degree of optic nerve involvement, or individual patient characteristics, may play a more significant role in determining baseline visual function than the tumor’s specific location. Further research may be required to identify additional predictive factors that influence visual outcomes in patients with meningiomas near the optic nerve.

### Preoperative ophthalmic features

Patients reported preoperative vision loss with a duration ranging from 3 to 24 months, with a median duration of 9 months. The majority of patients in our cohort had either intact or worsened vision preoperatively, with a smaller group experiencing blindness.

The distribution of patients in each preoperative vision group was as follows:

- Intact vision: 42 patients
- Worsened vision: 30 patients
- Blindness: 7 patients

Statistical analysis revealed a significant correlation between the duration of visual impairment and the severity of eyesight loss. Specifically, patients with longer durations of vision loss were more likely to experience more severe visual deterioration. This finding suggests that prolonged compression or other factors contributing to visual impairment may lead to more significant and irreversible damage to the optic nerve. Further investigation into the underlying mechanisms of this correlation could help inform strategies for early intervention and improve outcomes in patients with optic nerve-associated meningiomas.

### Tumor size and duration of visual impairment

Table 1 details the correlation between tumor size and the duration of visual loss. Patients with tumors less than 3 cm had a mean duration of visual impairment of 8.1 months (SD 5.2, median 6.0 months, range 4.0–24.0 months). Those with tumors measuring 3–5 cm had a mean duration of 8.8 months (SD 3.2, median 9.0 months, maximum 13.0 months), while patients with tumors larger than 5 cm showed a mean duration of 10.8 months (SD 5.4, median 12.0 months, maximum 16.0 months). Although the overall *p*-value was 0.095 (not reaching conventional significance), these data suggest that increasing tumor size may be associated with a more prolonged period of visual disturbance.

**Table 1:** Correlation between tumor size and vision loss duration.

	Tumor size						*p*
	< 3 cm (*n* = 31)		3–5 cm (*n* = 33)		> 5 cm (*n* = 15)		
	Mean	SD	Mean	SD	Mean	SD	
Vision loss duration in months	8.1	5.2	8.8	3.2	10.8	5.4	0.095

### Preoperative visual function and optic nerve involvement

Preoperative visual status was examined in relation to the tumor’s interaction with the optic nerve, which was categorized into dislocation, compression, intimate contact, and encasement. In the dislocation group (*n* = 9), 44.4% of patients had intact vision and 55.6% had worsened vision, with no cases of blindness. In the compression group (*n* = 31), 58.1% maintained intact vision, 35.5% exhibited worsened vision, and 6.5% were blind. Notably, patients in the intimate contact group (*n* = 18) had the most favorable preoperative outcomes, with 83.3% retaining intact vision, 11.1% showing deterioration, and only 5.6% being blind. In stark contrast, in the encasement group (*n* = 21), only 23.8% had intact vision, while 57.1% had worsened vision and 19.0% were blind. This distribution was statistically significant (*p* = 0.006), indicating that greater optic nerve involvement is associated with poorer preoperative visual function (Table 2).

**Table 2:** Correlation between preoperative vision and relationship with the ON.

*p* = 0.006		Preoperative vision			Total
		Intact *n* (%)	Worsened *n* (%)	Blindness *n* (%)	
Relationship with the ON	Dislocation	4 44.4%	5 55.6%	0 0.0%	9
	Compression	18 58.1%	11 35.5%	2 6.5%	31
	Intimate contact	15 83.3%	2 11.1%	1 5.6%	18
	Encasement	5 23.8%	12 57.1%	4 19.0%	21
Total		42 53.2%	30 38.0%	7 8.9%	79

### Postoperative visual outcomes relative to optic nerve involvement

Postoperative visual status varied considerably with the type of optic nerve involvement. For patients with dislocation (*n* = 9), 33.3% achieved intact vision after surgery, 33.3% showed improvement, 22.2% experienced further deterioration, and 11.1% remained unchanged. In the compression group (*n* = 31), 54.8% attained intact vision, 19.4% improved, 6.5% deteriorated, and 19.4% remained stable. Patients in the intimate contact group (*n* = 18) fared best postoperatively, with 77.8% achieving intact vision, 5.6% further improving, 5.6% worsening, and 11.1% showing no change. Conversely, in the encasement group (*n* = 21), only 19.0% achieved intact vision, 19.0% improved, 23.8% deteriorated, and 38.1% remained stable. The association between optic nerve involvement and postoperative visual outcome was statistically significant (*p* = 0.017) (Table 3).

**Table 3:** Correlation between postoperative vision and relationship with the ON.

*p* = 0.017		Postoperative vision				Total
		Intact *n* (%)	Improved *n* (%)	Worsened *n* (%)	Stable *n* (%)	
Relationship with the ON	Dislocation	3 33.3%	3 33.3%	2 22.2%	1 11.1%	9
	Compression	17 54.8%	6 19.4%	2 6.5%	6 19.4%	31
	Intimate contact	14 77.8%	1 5.6%	1 5.6%	2 11.1%	18
	Encasement	4 19.0%	4 19.0%	5 23.8%	8 38.1%	21
Total		38 48.1%	14 17.7%	10 12.7%	17 21.5%	79

### Visual field defects

In our study group, visual field defects were evaluated using Goldmann perimetry and integrated into a composite visual impairment score according to established guidelines. The predominant defect observed was temporal hemianopsia, which was present in approximately 60% of patients.

Among those with unilateral field deficits, 75% exhibited a localized temporal loss, whereas patients with bilateral involvement frequently demonstrated more extensive field constriction. Numerical scoring was applied—where, for instance, a monocular quadrant defect was assigned a score of 5, bilateral quadrant anopsia a score of 14, and bilateral hemianopsia a score of 22—to facilitate precise quantitative comparisons over time.

Statistical analysis revealed a significant correlation between the severity of the visual field loss and the duration of preoperative symptoms, suggesting that prolonged optic nerve compression was associated with more extensive field deficits. Moreover, patients with milder preoperative field impairments tended to show greater postoperative improvement, underscoring the prognostic value of early surgical decompression in preserving and enhancing visual function.

### Visual acuity and duration of visual impairment

The correlation between the duration of visual impairment and visual acuity was assessed preoperatively. The 42 patients with intact vision had a mean duration of 7.0 months, while patients with worsened vision (*n* = 30) also averaged 7.0 months (SD 3.2, median 7.0, maximum 15.0 months). In contrast, patients who were blind (*n* = 7) had a significantly longer mean duration of 13.7 months (SD 5.1, median 9.0, maximum 24.0 months). This difference was statistically significant (*p* = 0.009), suggesting that a prolonged period of visual impairment adversely affects visual acuity (Table 4).

**Table 4:** Correlation between visual acuity and duration of visual impairment.

	Visual acuity						*p*
	Intact (*n* = 42)		Worsened (*n* = 30)		Blindness (*n* = 7)		
	Mean	SD	Mean	SD	Mean	SD	
Duration of visual impairment	7.0		7.5	3.2	13.7	5.1	0.009

### Postoperative visual acuity and duration of visual impairment

Postoperative analysis showed a similar trend. The patients with intact vision postoperatively had a mean preoperative impairment duration of 7.0 months. Patients who experienced improvement (*n* = 13) had a mean duration of 6.8 months (SD 3.2, median 6.0, maximum 15.0 months). In contrast, those whose vision worsened (*n* = 7) had a mean duration of 8.6 months (SD 2.9, median 4.0), and patients with stationary outcomes (*n* = 12) had a mean duration of 11.3 months (SD 5.2, median 4.0, maximum 11.0 months). The relationship was statistically significant (*p* = 0.0498), reinforcing that shorter durations of visual impairment are associated with better postoperative visual acuity.

We attempted to identify a statistically significant cutoff point for determining the time threshold for visual improvement after surgery, based on the duration of symptoms. The optimal cutoff value for predicting visual deterioration is 8.5 months, as determined by the Youden’s J statistic, which indicates the point where the sum of sensitivity and specificity is maximized (see table). The sensitivity (SE) at this cutoff is 1, and the specificity (SP) is 0.692. This test is highly sensitive, meaning it is very good at correctly identifying patients with visual deterioration, but it is less specific, meaning there is a higher chance of false positives.

This cutoff value helps in predicting whether patients with symptoms lasting longer than 8.5 months are more likely to experience worsened vision postoperatively, though the trade-off is that the test may incorrectly classify some patients as at risk for deterioration despite having no worsening.

**Figure 1:**
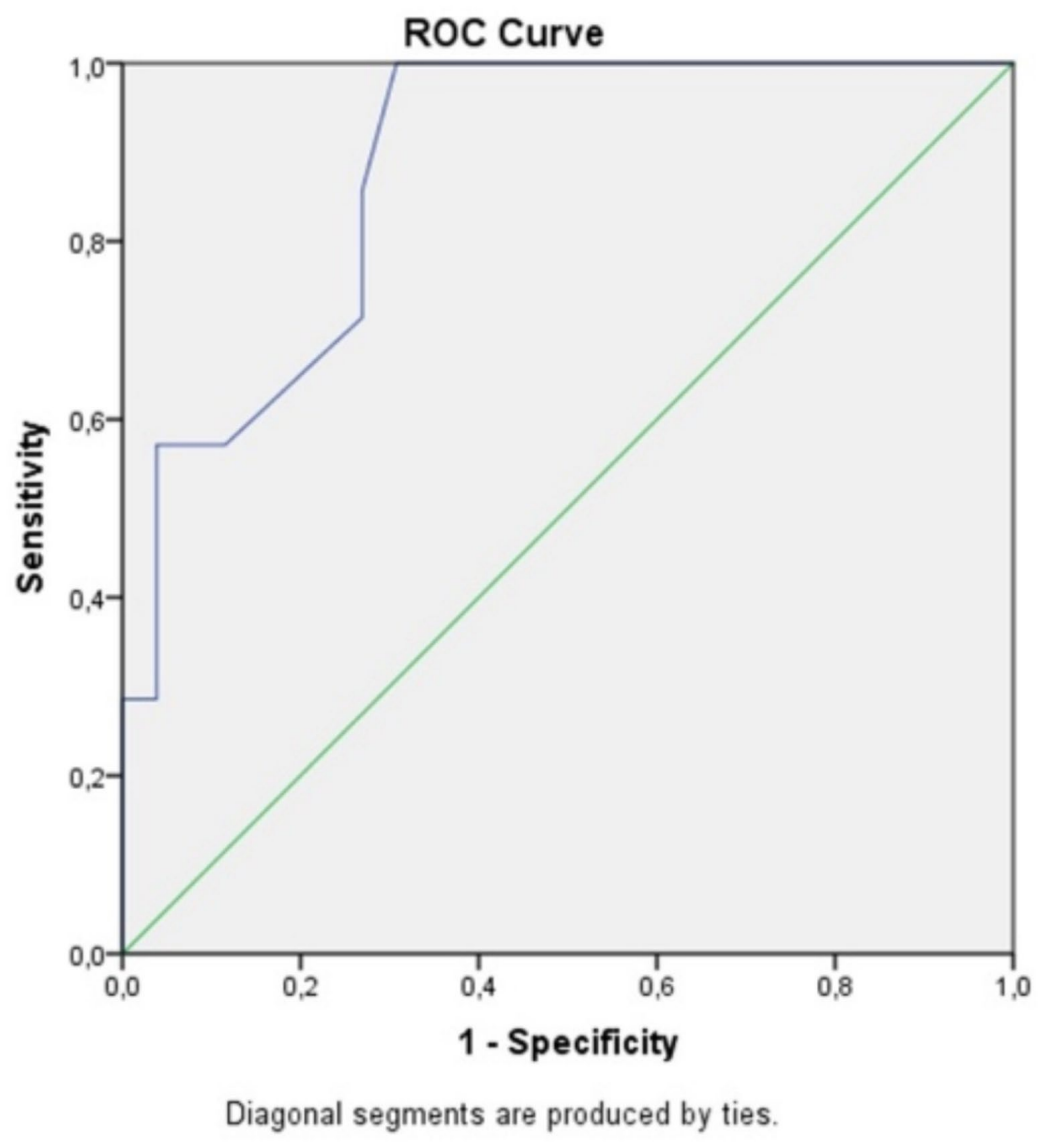
ROC curve comparing specificity and sensitivity for postoperative visual acuity in correlation to the symptoms duration.

The AUC (Area Under the Curve) of 0.882 indicates that the test discriminates very well. An AUC value close to 1 suggests excellent discriminatory power, meaning the test is highly effective at distinguishing between patients who will experience visual deterioration and those who will not. In this case, an AUC of 0.882 demonstrates that the test performs well in predicting visual outcomes based on the duration of symptoms before surgery, with a high degree of accuracy. This further supports the test’s usefulness in clinical decision-making (Figure 1).

## Discussion

This study offers valuable insights into the impact of meningiomas that compress the optic nerve (ON) on visual function, focusing on key factors such as tumor size, location, and the duration of preoperative visual impairment. The analysis highlights several critical trends in these variables, although some did not reach statistical significance, suggesting the need for further research and refinement of predictive models.

One of the notable trends observed in the data is the potential correlation between tumor size and the duration of vision impairment. Although this relationship did not achieve statistical significance, the data suggest that as tumor size increases, the duration of visual impairment may also tend to increase. This trend is consistent with the understanding that larger tumors often exert more pressure on the optic nerve, leading to a higher likelihood of progressive optic neuropathy. Larger tumors may cause more extensive and long-term damage to the optic nerve fibers, thus resulting in prolonged vision loss (11, 12).

These findings reinforce the clinical importance of early detection and intervention in patients with meningiomas near the optic nerve. Early identification of tumors, particularly larger ones, is crucial in preventing irreversible visual damage. Timely surgical or therapeutic interventions could potentially limit the extent of optic nerve compression and reduce the risk of severe or permanent vision loss. Furthermore, these results suggest that careful monitoring of tumor growth and visual function should be a priority for clinicians managing patients with meningiomas, particularly in cases where larger tumors are present or where vision impairment is already evident at the time of diagnosis (13, 14).

Interestingly, tumor location itself did not statistically impact preoperative or postoperative visual function, despite the known anatomical variations in optic nerve compression across different intracranial locations. This lack of correlation is notable, as one might expect certain tumor locations to have a more pronounced effect on visual function due to their proximity to critical structures like the optic nerve and optic chiasm. Several factors may explain this finding, including the relatively small sample size, the heterogeneity of tumor characteristics within each location group, or the complex nature of optic nerve compression, which involves multiple variables beyond just tumor location (15, 16).

This observation highlights the need for larger-scale studies with more comprehensive data to better understand the location-specific effects of meningiomas on visual outcomes (9). Larger cohorts may provide more robust insights into how different anatomical locations influence the severity and progression of visual impairment, allowing for more accurate predictions and improved management strategies for patients with meningiomas near the optic nerve (17).

The study revealed that the duration of preoperative visual impairment significantly correlated with the severity of visual function, with prolonged impairment being associated with worsened vision preoperatively. This finding supports existing research and emphasizes that the timing of symptom onset plays a crucial role in predicting visual outcomes (18). Patients who experienced shorter durations of vision loss (less than 8.5 months) tended to have better postoperative recovery, suggesting that surgical intervention may be most effective when performed shortly after the onset of visual symptoms.

Clinically, this 8.5-month threshold may help guide decisions on the timing of surgical intervention, especially in patients presenting with early or borderline symptoms. While the ROC analysis showed excellent sensitivity (1.0), its moderate specificity (0.692) indicates a risk of overtreatment in some cases. However, this trade-off may be acceptable when weighed against the potential for irreversible visual loss associated with delayed surgery. As such, this finding supports the clinical prioritization of timely intervention in patients with progressive optic nerve compression.

These findings suggest that earlier detection and intervention may be beneficial, although larger, prospective studies are needed to confirm these associations This could potentially inform future guidelines for treatment timing in patients with meningiomas that compress the optic nerve (ON) (19).

Interestingly, postoperative visual outcomes did not correlate with tumor size, location, or the duration of preoperative symptoms, which underscores the complexity of predicting visual recovery in this patient population (20). While factors such as tumor size and location intuitively seem to impact visual outcomes, the lack of a direct correlation in this study suggests that other variables, such as the extent of optic nerve damage, surgical technique, or individual patient factors, may play a more prominent role. However, a trend was observed indicating that shorter preoperative impairment durations may increase the likelihood of postoperative improvement. This suggests that early surgical intervention could improve the chances of visual recovery, further emphasizing the importance of timely treatment.

These findings highlight the multifactorial nature of visual outcomes following surgery for ON-compressing meningiomas and suggest that prompt surgical intervention remains a key factor in optimizing prognosis for patients with optic nerve involvement. Future studies with larger patient cohorts and more detailed assessments could help clarify the complex interplay of variables that influence visual recovery and help refine strategies for the management of these tumors (20, 21).

## Conclusion

Our findings underscore the importance of tumor size and, particularly, the duration of preoperative vision loss as key factors influencing visual outcomes in patients with optic nerve-compressing meningiomas. While not all observed trends reached statistical significance, this study offers valuable insights into the complex interplay of variables that contribute to visual recovery. Our findings suggest that the duration of vision loss prior to surgery may be an important factor in visual prognosis, warranting further study to determine whether earlier intervention improves outcomes. Although some factors, such as tumor size and location, did not show a direct correlation with visual recovery, their potential role in conjunction with other variables warrants further investigation.

This study provides a foundation for understanding the factors associated with visual recovery and offers direction for future research aimed at refining prognostic models for patients with optic nerve compression due to meningiomas. Given the complexity of visual outcomes, future studies with larger sample sizes and more detailed subgroup analyses will be crucial to confirm these associations and to better predict which patients are most likely to benefit from surgical intervention. Additionally, these findings may help guide the development of more tailored therapeutic strategies, optimizing the timing of surgery and other interventions to preserve or improve vision in affected patients.

Further research, particularly with multicentre studies, could provide more robust evidence, helping clinicians make more informed decisions and improving patient care by refining the parameters that influence visual outcomes in this patient population.

## What is already known on this topic

Prior studies have shown that optic nerve compression by meningiomas leads to progressive visual loss, with factors such as tumor size, location, and duration of preoperative impairment thought to influence outcomes. However, the precise predictors of visual recovery remain unclear.

## What this study adds

This study demonstrates that the duration of preoperative visual impairment is a critical determinant of postoperative visual recovery, with shorter impairment durations correlating with better outcomes. Notably, tumor size and location did not significantly impact recovery, underscoring the importance of early intervention.

## How this study might affect research, practice or policy

These findings support the need for prompt diagnosis and early surgical management in patients with optic nerve–compressing meningiomas, potentially informing clinical guidelines and future research aimed at refining prognostic models for visual outcomes.

## Data Availability

The raw data supporting the conclusions of this article will be made available by the authors, without undue reservation.
